# Pulmonary arteriovenous fistula secondary to historic thoracic shrapnel injury: presenting features and interventional approach

**DOI:** 10.1186/s42155-024-00494-y

**Published:** 2025-04-14

**Authors:** Kate Chiswell, Ahmed Al-Hindawi, Richard Dunn, Richard Waugh, Rachael Cordina

**Affiliations:** 1 Royal Prince Alfred Hospital, 50 Missenden Road, Camperdown, NSW Australia; 2 Department of Radiology, Royal Prince Alfred Hospital, Sydney, Australia; 3 Bankstown-Lidcombe Hospital, Sydney, Australia; 4 The University of Sydney, Sydney, Australia

**Keywords:** Bubble echocardiography, Cyanotic heart disease, Hypoxemia, Imaging, Occlude, Shortness of breath

## Abstract

**Background:**

A chronic pulmonary arteriovenous fistula (PAVF) post penetrating thoracic injury is rare; this case demonstrates the presentation, diagnostic implications and a successful endovascular approach to close it.

**Case presentation:**

We report the case of a rare pulmonary arteriovenous fistula in a 50-year-old man secondary to historic thoracic shrapnel injury. He had a positive bubble study on echocardiography; CT pulmonary angiogram identified a pulmonary arteriovenous fistula. He underwent successful repair with an endovascular approach.

**Conclusion:**

PAVF can result from penetrating chest trauma. An intra-pulmonary shunt should be suspected in the setting of hypoxaemia refractory to oxygen therapy and when there is no evidence of an intra-cardiac shunt. Endovascular intervention can facilitate definitive treatment by employing a plug-in-stent technique.

**Supplementary Information:**

The online version contains supplementary material available at 10.1186/s42155-024-00494-y.

## Background

Pulmonary arteriovenous fistulae are uncommon [1] and mostly congenital or the result of genetic issues, such as hereditary haemorrhagic telangiectasia and Fanconi syndrome [2]. Acquired causes include hepatic cirrhosis and pulmonary schistosomiasis [1]. It is very rare to be associated with penetrating injuries, including chest trauma [2, 3], likely because of the high mortality rate associated with culprit trauma and subsequent surgery, as well as the low pressure gradient between pulmonary arteries and veins [1].

A chronic pulmonary arteriovenous fistula (PAVF), post penetrating thoracic injury is rare; this case demonstrates the clinical presentation, diagnostic implications and a successful endovascular approach to close it.

## Case presentation

A 50-year-old man with a history of shrapnel injury to the thorax in the 1990s was referred to our centre with worsening unexplained dyspnoea, cyanosis and hypoxemia over several years, refractory to high-flow oxygen. Prior radionuclide shunt study was suggestive of a right-to-left shunt. On presentation, his oxygen saturations were 87% on room air with clubbing and central cyanosis. An arterial blood gas on room air demonstrated pO2 of 49 mmHg and arterial saturations of 84% without improvement on high flow oxygen. His serum haemoglobin was elevated in the context of erythrocytosis. We noted shrapnel wounds on his upper right anterior thorax and suspected that he had a trauma-related intrapulmonary shunt (Supplementary Fig. 1).

He had a history of mild chronic obstructive pulmonary disease (COPD) based on lung function testing and a high-resolution chest CT. He had a 60-pack year smoking history. His other relevant background included a previous left renal carcinoma with radical nephrectomy in 2020, previous deep vein thrombosis complicated by pulmonary embolus in 2017 and H1N1 influenza in 2019 that required a brief intensive care admission.

The relevant differential diagnoses included an intracardiac and non-traumatic intrapulmonary shunts.

‘Bubble contrast’ echocardiography demonstrated a torrentially positive bubble study within three cardiac cycles, a small patent foramen ovale without evidence on colour flow of right-to-left shunting and normal cardiac chamber size and function. Right heart catheterisation performed prior to referral demonstrated normal pulmonary vascular resistance. We noted a large right hilar mass suspicious for a vascular abnormality on a CT chest from 2019. A CT pulmonary angiogram (CTPA) was performed to characterize this mass. An 8 mm-diameter metallic density in the right lower lobe of the lung was evident that was also visible on chest radiography. In the arterial phase, a marked focal dilatation was identified in the right superior pulmonary vein, which was in communication with the right lower lobe A6 superior segment pulmonary artery (Supplementary Fig. 1). This confirmed the presence of a large systemic venous to pulmonary shunt, likely the result of shrapnel injury many years earlier.

We proceeded to formal angiography with view to fistula closure. A 10Fr catheter, later upsized to a 12Fr catheter, was introduced by right femoral vein access. A 0.014″ microwire was passed into the feeding vessel beyond the aneurysm neck, followed by a 0.018″ stability microwire that was passed into the right lower lobe artery. An Abbott 8.6 mm x 40 mm stent was positioned across the aneurysm neck. Following this, a 13 mm microvascular plug (MVP-9Q from Reverse Medical) was deployed within the stent and after approximately five minutes, there was no further filling of the fistula from the pulmonary arterial side (Supplementary Fig. 2). There were no immediate complications from this procedure.

On day one post device insertion, oxygen saturations on room air had substantially improved to 94%. CTPA demonstrated a subtle residual contrast blush arising near the origin of the stent, visible within the enlarged right superior pulmonary vein. There was no convincing contrast opacification within the stent, or in the vessel arising from the distal aspect of the stent. He was commenced on aspirin and clopidogrel for 3 months to prevent thrombus formation in the residual, now blind-ending large vascular structures that communicated with the pulmonary venous system that were expected to regress over time. He was treated for mild hospital-acquired pneumonia with amoxicillin-clavulanic acid prior to discharge.

A planned three-month follow-up contrast CT chest demonstrated a small amount of persistent flow through the defect in the setting of oxygen saturations of 93–95%. A second procedure was undertaken to obliterate the shunt. The right common femoral vein was accessed and a 55 cm 6Fr sheath was placed. 5Fr pigtail catheter right pulmonary angiograms demonstrated a residual shunt with flow around the previously deployed stent and plug. A microcatheter was advanced aside the stent into a small branch arising from the proximal aspect of the stent and several detachable coils (AZUR-CX coils from Terumo Interventional Systems) were deployed from the small branch artery proximally, around the taper of the plug within the stent, and up to the origin of the right A6 pulmonary artery (Supplementary Fig. 3). This resulted in successful occlusion of the shunt. On follow-up CT pulmonary angiography one month later, there was no residual shunt with obliteration of the flow-related pulmonary venous varix. Three months later, his cardiovascular examination and echocardiography were unremarkable. Oxygen saturations were 99% on room air and he had no limitation on exercise capacity.

## Discussion

As mentioned, it is rare for PAVFs to be associated with penetrating chest injuries. In similar cases reported, presentation was often delayed, with some patients remaining asymptomatic for many years after the initial culprit trauma [1–4]. Symptoms reported include breathlessness, chest pain, hypoxia, clubbing, haemoptysis and heart failure [1]. Interestingly, despite the time between the traumatic event and presentation, our patient had no hemoptysis, heart failure or evidence of sequelae, such as brain abscess or paradoxical emboli. It is therefore important to consider a PAVF as a diagnosis in this setting when there is a history of penetrating chest trauma and no overt symptoms aside from a reduced oxygen saturation.

As part of the work-up for unexplained cyanosis, echocardiography is routinely performed [5]. Classical teaching suggests that early bubbles are consistent with an intracardiac shunt rather than an intrapulmonary one. While the bubble contrast echocardiography was positive in our case, the lesion was very large thus the systemic venous system bubbles were appearing earlier than would usually be expected. Thus, relying on the timing of bubbles in a bubble study may not be a reliable way to differentiate between intra-pulmonary and intra-cardiac shunts. CTPA is considered the gold standard for defining vascular anatomy of PAVFs for potential embolisation [6], and is essential in the early diagnosis of PAVFs post penetrating injury.

Embolotherapy, with coils, vascular plugs and occluder devices, is usually the preferred treatment given lower morbidity and mortality compared with surgical approaches in the current era [6]. Surgical repair may be required in the setting of traumatic multi-vessel involvement [1], initial failed embolisation [2] or large fistula size [7]. Other cases have described the successful closure of large PAVFs with large septal occluder devices, such as an Amplatzer “Cribiform” Occluder [8–10]. In our case, an uncovered stent was positioned across the aneurysm neck due to the proximity of the fistula to the main right lower lobar pulmonary artery. The stent acted as a scaffold for the plug, which was deployed within this stent, thus holding it in place and helping to prevent migration further into the pulmonary arterial supply. This was important to prevent non-target embolisation because of the proximal nature of the fistula. A small residual shunt on CT was obliterated by the deployment of several coils around the taper of the plug within the stent, which was thought to be the cause of incomplete occlusion. To our knowledge, we are the first to report a plug-in-stent approach in this setting.

Two months following his second procedure the patient presented to hospital with a trash foot that resolved spontaneously. This was likely secondary to an arterial embolic event related to a thrombus that had developed in the occluded fistula. He was commenced on rivaroxaban for an additional three months to allow for endothelialisation of the intravascular material. He remains well, now over 12 months following his initial procedure.

## Conclusions

PAVF can result from penetrating chest trauma. An intra-pulmonary shunt should be suspected in the setting of hypoxaemia refractory to oxygen therapy and when there is no evidence of an intra-cardiac shunt. Endovascular intervention may allow definitive treatment, even when the defect is unsuitable for vascular plugs and coils alone, by employing a plug-in- stent technique. 

## Supplementary Information

Supplementary Material 1: Supplementary Fig. 1. Axial maximum intensity projection (A) and Coronal (B) contrast enhanced CT demonstrating the large varix (arrow heads) of the right superior pulmonary vein just distal to the fistula (arrow) with the right lower lobe A6 superior segment pulmonary artery (asterisk). 1(C) demonstrates the shrapnel injury on clinical examination. Supplementary Fig. 2: (A) Stent within the right A6 pulmonary artery branch (arrow) across the neck of the fistula (arrowhead). (B) Following expansion of the stent and deployment of a 13 mm microvascular plug within the stent (arrow) demonstrating no visible filling through the fistula. (C&D) Post treatment oblique curved and coronal contrast enhanced CT demonstrates enhancement within the varix (asterisk) with the plug-in-stent across the neck (short arrow). A thin jet of contrast (long arrow) is seen filling the varix. Supplementary Fig. 3: (A) Right interlobar pulmonary artery angiogram with plug-in-stent in situ (arrow) but there is opacification of the varix (asterisk), found to be supplied by the origin of a small branch artery arising just proximal to the stent. (B) Detachable coils (arrowheads) deployed distally within the branch artery and packed against the proximal opening of the stent around the tapered end of the microvascular plug. (C&D) Post second treatment axial and coronal post contrast CT demonstrates the plug-in-stent (arrow) and coils (arrowheads) with resulting obliteration of the varix and fistula.

## Data Availability

Not applicable.

## Abbreviations

COPD: Chronic obstructive pulmonary disease
CTPA: CT Pulmonary angiogram
PAVF: Pulmonary arteriovenous fistula
MVP: Microvascular plug

## References

[1] Williams AB, Lax LG. Delayed presentation of a rare and complex multi-vessel pulmonary arteriovenous fistula following penetrating chest-wall trauma. J Surg Case Rep. 2021;2021(2):rjaa605. 10.1093/jscr/rjaa605. 33643606 10.1093/jscr/rjaa605 PMC7896838

[2] Azimi-Ghomi O, Ramirez M, Brummund D, et al. Traumatic pulmonary arteriovenous malformation presenting as spontaneous hemothorax. Cureus. 2021;13(6): e16072. 10.7759/cureus.16072. 34345554 10.7759/cureus.16072 PMC8324253

[3] Asensio JA, Dabestani PJ, Miljkovic SS, et al. Traumatic penetrating arteriovenous fistulas: a collective review. Eur J Trauma Emerg Surg. 2021;48:775–89. 10.1007/s00068-020-01574-z. 33386864 10.1007/s00068-020-01574-z

[4] Manganas C, Iliopoulos J, Pang L, Grant PW. Traumatic pulmonary arteriovenous malformation presenting with massive hemoptysis 30 years after penetrating chest injury. Ann Thorac Surg. 2003;76(3):942–4. 10.1016/s0003-4975(03)00527-7. 12963241 10.1016/s0003-4975(03)00527-7

[5] Gossage JR. Therapeutic approach to adults with pulmonary arteriovenous malformations. In: Mandel J, Finlay G, editors. UptoDate. 2020. Available from: https://www.uptodate.com/contents/therapeutic-approach-to-adult-patients-with-pulmonary-arteriovenous-malformations?sectionName=EMBOLOTHERAPY&search=pulmonary%20AVM&topicRef=8268&anchor=H148211957&source=see_link#H148211957. [cited 2022 Feb 27].

[6] Abdel Aal AK, Eason J, Moawad S, et al. Persistent pulmonary arteriovenous malformations: percutaneous embolotherapy. Curr Probl Diagn Radiol. 2018;47(6):428–36. 10.1067/j.cpradiol.2017.09.006. 29103837 10.1067/j.cpradiol.2017.09.006

[7] Ploch PJ, Datta S, Thompson JH, Raghavendran K. Posttraumatic pulmonary arteriovenous fistula: is resection the procedure of choice? A case report and review of literature. J Trauma. 2009;66(2):554–7. 10.1097/01.ta.0000221550.24390.6e. 18349712 10.1097/01.ta.0000221550.24390.6e

[8] Healan S, Varga P, Shah AP. Percutaneous transcatheter closure of a large systemic to pulmonary venous fistulae in an adult patient after extracardiac fontan. Catheter Cardiovasc Interv. 2015;86(3):472–5. 25708733 10.1002/ccd.25898

[9] Mauri S, Fuller B, Szymanski T, Wang DD, Alaswad K. Percutaneous closure of post-traumatic pulmonary arteriovenous fistula. J Am Coll Cardiol. 2019;73(9_Supplement_1):2748.

[10] Liu XH, Yang JM. Management of paradoxical embolism in a patient with coexisting patient foramen ovale and masked pulmonary arteriovenous fistula: a case report. Medicine (Baltimore). 2020;99(15): e19507. 10.1097/MD.0000000000019507. 32282700 10.1097/MD.0000000000019507 PMC7220135

