# Supplementary material for: Pulmonary arteriovenous fistula secondary to historic thoracic shrapnel injury: presenting features and interventional approach
Source: CVIR Endovasc. 2025 Apr 14;8:30. doi: 10.1186/s42155-024-00494-y (PMC11996724; doi:10.1186/s42155-024-00494-y)
Supplement: Supplementary file 1 — Supplementary Material 1: Supplementary Fig. 1. Axial maximum intensity projection (A) and Coronal (B) contrast enhanced CT demonstrating the large varix (arrow heads) of the right superior pulmonary vein just distal to the fistula (arrow) with the right lower lobe A6 superior segment pulmonary artery (asterisk). 1(C) demonstrates the shrapnel injury on clinical examination. Supplementary Fig. 2: (A) Stent within the right A6 pulmonary artery branch (arrow) across the neck of the fistula (arrowhead). (B) Following expansion of the stent and deployment of a 13 mm microvascular plug within the stent (arrow) demonstrating no visible filling through the fistula. (C&D) Post treatment oblique curved and coronal contrast enhanced CT demonstrates enhancement within the varix (asterisk) with the plug-in-stent across the neck (short arrow). A thin jet of contrast (long arrow) is seen filling the varix. Supplementary Fig. 3: (A) Right interlobar pulmonary artery angiogram with plug-in-stent in situ (arrow) but there is opacification of the varix (asterisk), found to be supplied by the origin of a small branch artery arising just proximal to the stent. (B) Detachable coils (arrowheads) deployed distally within the branch artery and packed against the proximal opening of the stent around the tapered end of the microvascular plug. (C&D) Post second treatment axial and coronal post contrast CT demonstrates the plug-in-stent (arrow) and coils (arrowheads) with resulting obliteration of the varix and fistula. [file 42155_2024_494_MOESM1_ESM.docx]

**Appendix and Figures**

C


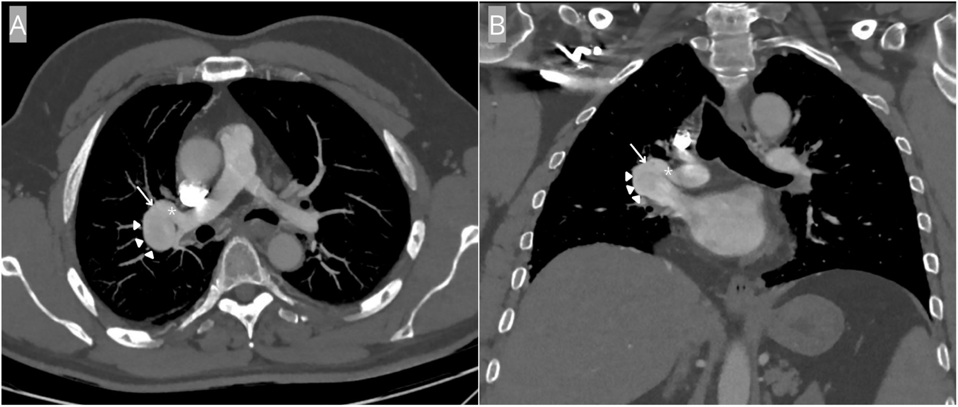
**
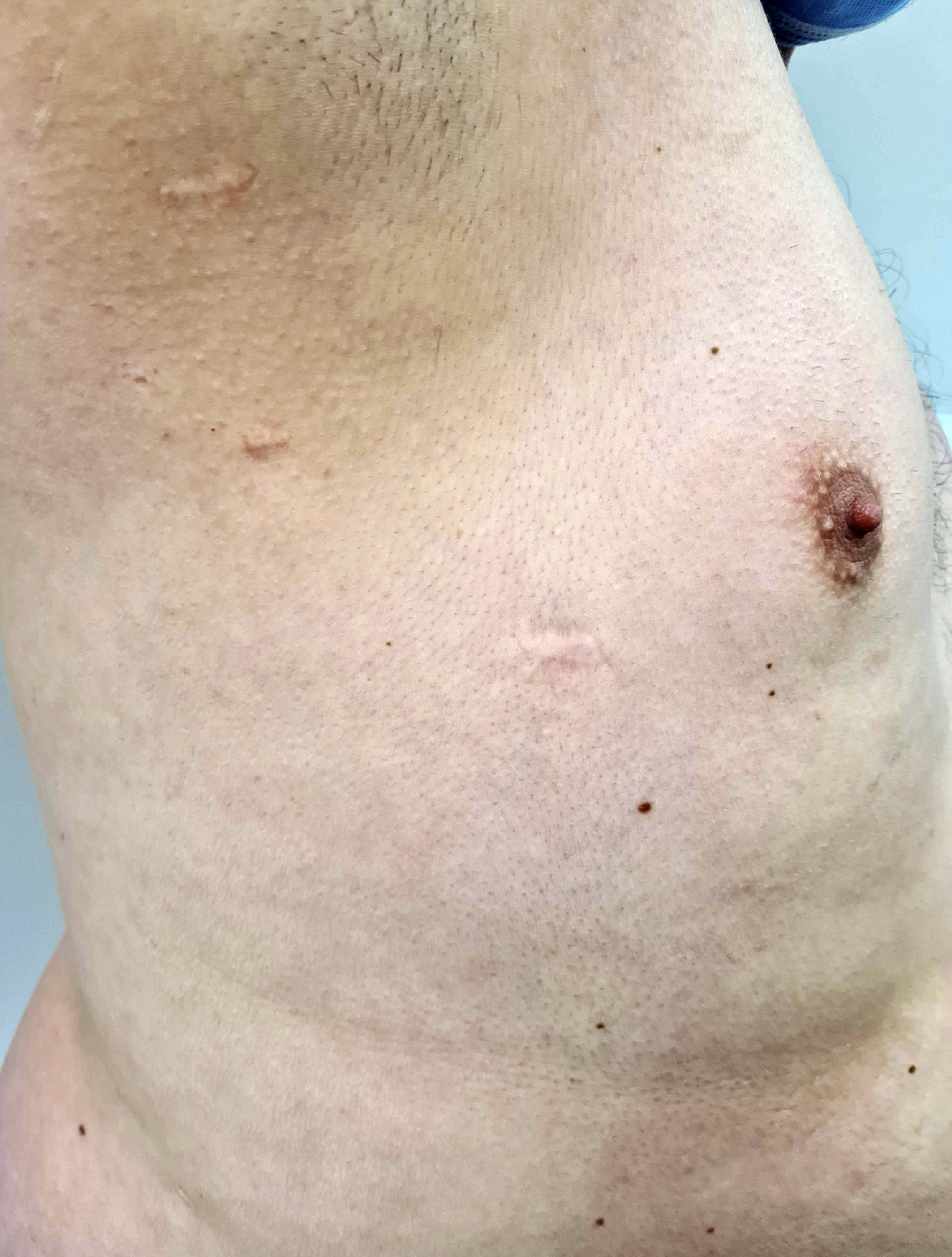
**

Supplementary Figure 1: Axial maximum intensity projection (A) and Coronal (B) contrast enhanced CT demonstrating the large varix (arrow heads) of the right superior pulmonary vein just distal to the fistula (arrow) with the right lower lobe A6 superior segment pulmonary artery (asterisk). 1(C) demonstrates the shrapnel injury on clinical examination.


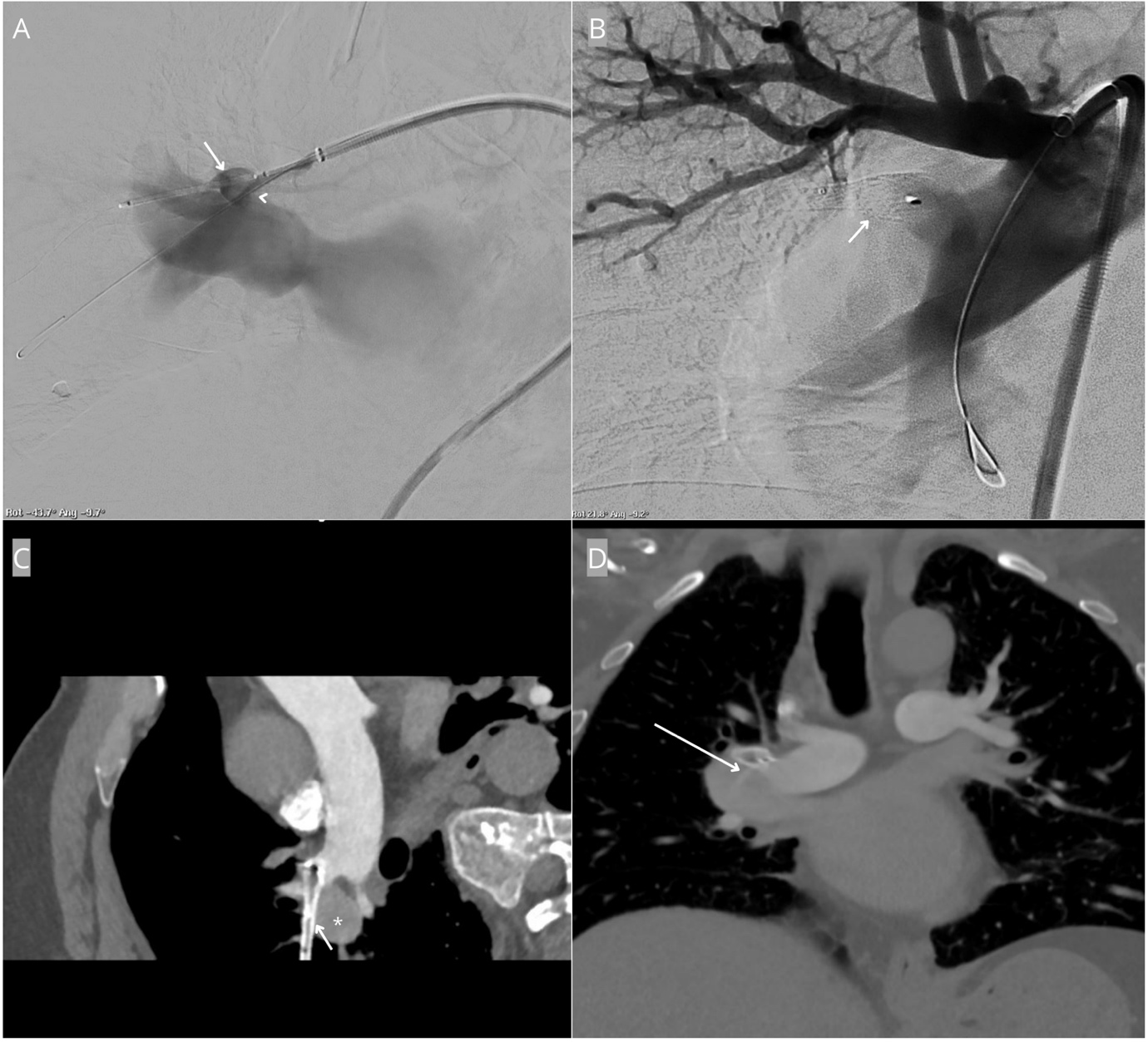


Supplementary Figure 2: (A) Stent within the right A6 pulmonary artery branch (arrow) across the neck of the fistula (arrowhead). (B) Following expansion of the stent and deployment of a 13mm microvascular plug within the stent (arrow) demonstrating no visible filling through the fistula. (C&D) Post treatment oblique curved and coronal contrast enhanced CT demonstrates enhancement within the varix (asterisk) with the plug-in-stent across the neck (short arrow). A thin jet of contrast (long arrow) is seen filling the varix.


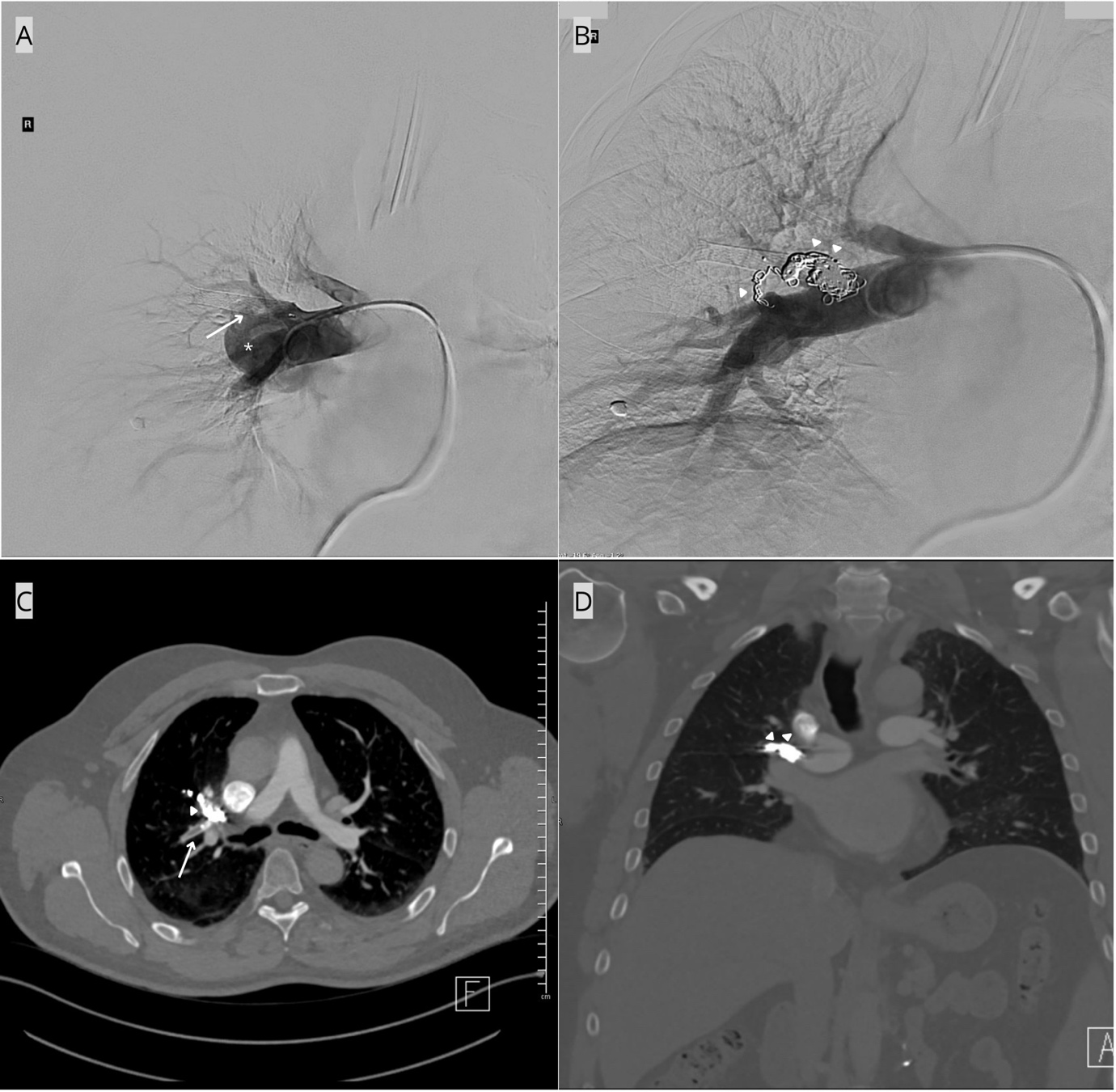


Supplementary Figure 3: (A) Right interlobar pulmonary artery angiogram with plug-in-stent in situ (arrow) but there is opacification of the varix (asterisk), found to be supplied by the origin of a small branch artery arising just proximal to the stent. (B) Detachable coils (arrowheads) deployed distally within the branch artery and packed against the proximal opening of the stent around the tapered end of the microvascular plug. (C&D) Post second treatment axial and coronal post contrast CT demonstrates the plug-in-stent (arrow) and coils (arrowheads) with resulting obliteration of the varix and fistula.
